# Knockdown of NDUFC1 inhibits cell proliferation, migration, and invasion of hepatocellular carcinoma

**DOI:** 10.3389/fonc.2022.860084

**Published:** 2022-09-02

**Authors:** Fang Han, Junwei Liu, Hongwu Chu, Dan Cao, Jia Wu, Hong Fu, Anyang Guo, Weiqin Chen, Yingping Xu, Xiangdong Cheng, Yuhua Zhang

**Affiliations:** ^1^ The Cancer Hospital of the University of Chinese Academy of Sciences, Zhejiang Cancer Hospital, Institute of Basic Medicine and Cancer (IBMC), Chinese Academy of Sciences, Hangzhou, China; ^2^ Hepatobiliary and Pancreatic Surgery, Zhejiang Provincial People’s Hospital, Hangzhou Medical College, Hangzhou, China; ^3^ Department of Medicine, Qingdao University, Qingdao, China; ^4^ College of Food and Pharmacy, Zhejiang Ocean University, Zhoushan, China; ^5^ Hepatobiliary and Pancreatic Surgery Dept., Shaoxing Peoples’s Hospital, Shaoxing, China

**Keywords:** HCC, NDUFC1, senescence, NADH: ubiquinone oxidoreductase complexes I, prognosis

## Abstract

**Background:**

NADH: ubiquinone oxidoreductase subunit C1(NDUFC1) encodes a subunit of the Complex I, which may support the structural stability of Complex I and assist in its biogenesis. The expression and functional roles of NDUFC1 in hepatocellular carcinoma (HCC) remain unknown.

**Result:**

We knocked down the expression of NDUFC1 in HCC cell lines to explore the effects of NDUFC1 downregulation on HCC *in vitro*. MTT assay determined that downregulation of NDUFC1 significantly inhibited cell proliferation. Flow cytometry with (propidium iodide) PI staining indicated silencing of NDUFC1 arrested cell cycle of BEL-7404 cells at G2 phase and SK-HEP-1 cells at S/G2 phase. Annexin V-PI double staining and flow cytometric analysis showed that the downregulation of NDUFC1 significantly increased the population of apoptotic cells. Wound-healing assay and transwell assay indicated that the downregulation of NDUFC1 suppressed the migration and invasion of HCC cells. According to the detection of complex1 activity, we found that the activity of NDUFC1 silenced group decreased, whereas the content of ROS increased. Furthermore, combined with bioinformatics analysis of senescence-related genes, we found that the silence of NDUFC1 in HCC could induce senescence and inhibit autophagy. In addition, NDUFC1 could correlate positively with cancer-related pathways, among which the p53 pathways and the PI3K/Akt/mTOR pathways. Finally, NDUFC1 is high expression in HCC specimens. High NDUFC1 expression was associated with poor prognosis and was an independent risk factor for reduced overall survival (OS).

**Conclusions:**

Our study indicated, for the first time, that NDUFC1 is an independent risk factor for the poor prognosis of HCC patients. NDUFC1 may promote tumor progression by inhibiting mitochondrial Complex I and up-regulating ROS through multiple cancer-related and senescence-related pathways of HCC, including p53 pathways and PI3K/Akt/mTOR pathways. We suppose that NDUFC1 might be a potential target for the mitochondrial metabolism therapy of HCC.

## Introduction

Hepatocellular carcinoma (HCC) is one of the most common malignant tumors and the fourth leading cause of cancer-related death worldwide (1). The incidence of HCC continues to rise, and due to the high recurrence and intrahepatic metastasis rate, the prognosis of patients with HCC is poor (2). Early diagnosis is essential for improving patient prognosis. Thus, it is essential to discover the molecules associated with HCC for its therapy (3). Mitochondria play an important role in cell metabolism, and mitochondrial oxidative phosphorylation complexes are essential for the production of adenosine triphosphate (ATP) (4). In addition to providing energy, mitochondria also play an important role in regulating the cell cycle, cell apoptosis, Ca^2+^ homoeostasis, and Fe–S cluster biogenesis (5, 6). Complex I (NADH: ubiquinone oxidoreductase) is the first enzyme of the mitochondrial respiratory chain. It is composed of 45 subunits and exists in supercomplex forms with respiratory chain complexes III and IV, which generate proton motive force used to synthesize ATP (7, 8). Complex I dysfunction is associated with the progression of a variety of tumors (9–11). NADH: ubiquinone oxidoreductase subunit C1 (NDUFC1) encodes a 76 amino subunit protein of the Complex I, which may support the structural stability of Complex I, but not involved in catalysis (12). Currently, there is no research report on the relationship between NDUFC1 and HCC.

In addition, NDUFC1 constitutes the structure of mitochondrial Complex I, we started from the function of mitochondria itself and explored the changes of mitochondrial function. In addition, cell senescence is also an anti-tumor property that has been gradually discovered in recent years. Because NDUFC1 is located on mitochondrial Complex I, we examined the activity of Complex I and ROS levels. Furthermore, we measured a serious tumor-related phenotypes to explore its possible mechanism. The aim of this study was to further elucidate the role of NDUFC1 in HCC and the possible mechanisms.

## Materials and methods

### Cell lines

The HCC cell lines (SK-HEP-1 and BEL-7404) were cultured in a complete medium, Dulbecco’s modified Eagle’s medium (DMEM) (HyClone, Logan, UT, USA) containing 10% fetal bovine serum (FBS) (Weike Biochemical Reagent, Shanghai, China), 100 units/ml penicillin, and 100 μg/ml streptomycin (CA, USA). All cells were cultured at 37°C in humidified 5% CO_2_ atmosphere.

### Plasmids and transfection

For silencing NDUFC1 expression, three RNA interference target sequences were designed. Small hairpin RNA (shRNA) (10 μM) targeting to human NDUFC1 was synthesized based on selected target sequences by the Generay Biotech (Shanghai, China). We designed three target shRNA for NDUFC1, respectively, 5’-AAAGAAGAAATGGGCTGGAAT-3’, 5’-GTGGATCTATCTCATCAAACA-3’, 5’-ATCAAAGTTCTACGTGCGAGA-3’. The control scrambled shRNA was 5’- TTCTCCGAACGTGTCACGT-3’. Then, the combined optimal sequences of shRNAs targeting to NDUFC1 and control scrambled shRNA were cloned into the lentivirus vector (pBR-V108) to form constructed pBR-V108-shRNA plasmids. The shRNA vectors were then separately transfected into 293T cells using Lipofectamine 2000 (Invitrogen, Shanghai, China) to generate the respective lentiviruses. After 2 days of transfection, 293T cells were collected and concentrated for 3 days. 24 h after being seeded into six-well plates, BEL-7404, and SK-HEP-1 cells were incubated with NDUFC1-siRNA-lentivirus (Lv-shNDUFC1 group, pSLenti-U6-shRNA(NDUFC1)-CMV-EGFP-F2A-Puro-WPRE) or negative control lentivirus (Lv-shCtrl group, pSLenti-U6-shRNA(NC2)-CMV-EGFP-F2A-Puro-WPRE) in serum-free transfection medium (GIBCO, NY, USA) for 18 h, and then cultured in fresh complete DMEM for 72 h. After lentiviral infection, GFP expression was observed by fluorescence microscope to determine infection efficiency.

### qRT-PCR

The RNA was extracted by TRIzol (Invitrogen Cat. No.15596-026). The cDNA Synthesis Kit (TAKARA Cat. No. 639506) was used to synthesize cDNA. Real-time PCR was performed on ABI-7500 using UltraSYBR One Step RT-qPCR Kit (Sangon Biotech Cat. No. B639277-0100). The primers were listed as followed: NDUFC1 Forward: 5’-AGTGCGATCAAAGTTCTACGTG-3’ and Reverse: 5’-AGAAGACAGTGGTGCCCAAG-3’, Total length is 89 bp.

### Western blotting analysis

Whole-cell extracts were prepared as described previously (13). Equal amount (50 μg) of the protein was separated on 10% gradient SDS polyacrylamide gels at 50 V for 3 h. The protein was then transferred to polyvinylidene fluoride (PVDF) membranes (Millipore Cat.IPVH00010, Millipore) at 4°C, 300 mA for 2.5 h, and blocked with 5% non-fat milk for 1 h. Then, the membranes were subsequently incubated with the primary anti-NDUFC1 (Invitrogen, 1:1000 dilution) or GAPDH (Bioworld, 1:3000 dilution) and peroxidase-conjugated second antibody (Beyotime, Shanghai, 1:3000 dilution). The membranes were washed (3×) by TBST and incubated with enhanced chemiluminescent substrate (Amersham, uppsala, Sweden) and exposed to Hyperfilm (Kodak Image Station 440CF; Kodak). All primer-antibody and dilution rate were presented in **Supplementary Table 2**.

### Cell proliferation assay

For MTT assay, 5,000 cells per well Lentiviral-infected BEL-7404 and SK-HEP-1 cells were seeded in 96-well plates and cultured in a 5% CO_2_, 37˚C incubator. Every 24 h, one plate was taken randomly and 20 μl of MTT (5 mg/ml, Genview) was added to each well. After being incubated at 37°C for 4 h, 100 μl of DMSO (Shiyi, Shanghai, China) was added into each well. Then, the plates were measured at a wavelength of 490/570 nm. The values obtained are proportional to the amounts of viable cells.

### Cell cycle assay

Lentiviral infected BEL-7404 and SK-HEP-1 cells were seeded in 60-mm cell culture dishes (1 × 10^6^ cells per dish) and incubated at 37°C. After the cell confluence reached ~80%, the cells were harvested and washed in cold phosphate-buffered saline (PBS) and fixed with pre-cold 70% ethanol for 1 h. After being washed by pre-cold PBS and digested by RNase (TakaRa, Japan), the cells were stained with propidium iodide (PI) at 4°C in dark for 20 min. Then, the flow cytometer (Millipore, USA) was used to determine the cell cycle.

### Cell apoptosis assay

Lentiviral-infected BEL-7404 and SK-HEP-1 cells were seeded in six-well plates (2 × 10^6^ cells per well) and cultured for 48 h. Then, the cells were subjected to Annexin V-APC/PI double staining following the instruction of Apoptosis Assays Kit (eBioscience, California, USA). The percentage of apoptotic cells was analyzed by FACS caliber II sorter and Cell Quest FACS system (BD Biosciences, USA).

### Cell migration assay

Wound-healing testing was used to access the migration ability of cells. BEL-7404 (5 × 10^5^ per well) and SK-HEP-1 (5 × 10^5^ per well) cells were seeded into 96-well plates. Then, the cells were cultured in complete DMEM for 24 h to reach confluence. Similar-sized wounds were introduced to monolayer cells using a plastic 200 μl of pipette tip. The wounded cells were washed (3×) with PBS to remove cell debris and cultured at 37°C. The cell migration ability was assessed by using the fluorescence microscope (Olympus Fluoview FV1200) to monitor the speed of wound closure at 8 h, 24 h, and 48 h, respectively.

### Cell invasion assay

The invasion ability of cells was assessed by transwell assay with a Matrigel-coated membrane (BD Bioscience, Sparks, MD, USA). 5 × 10^5^ BEL-7404 or SK-HEP-1 cells were suspended in 200 μl of serum-free medium and seeded into the upper chamber. In the lower chamber, 600 μl of medium supplemented with 10% FBS were added as a chemoattractant. After incubating at 37°C for 16 h, the cells in the upper chamber were scraped off gently with cotton swab. The invaded cells on the lower surface were fixed in 4% paraformaldehyde for 15 min and stained with 0.1% crystal violet for 10 min. The number of invaded cells were observed and counted in three randomly selected high-power fields under light microscope (×200).

### Mitochondrial respiratory chain Complex I activity detection

In this experiment, we used mitochondrial respiratory chain Complex I activity detection kit (CAT.BC0510, Solarbio Shanghai), and cells were collected, the extract reagent was added, homogenized on ice with a homogenizer, and centrifuged at 600*g* at 4°C for 10 min. The supernatant was transferred to another centrifugal tube and centrifuged at 11,000*g* at 4°C for 15 min. Complex I with mitochondrial leakage in the supernatant was taken and 400 μl of extraction reagent was added to the precipitate. Ultrasonic crushing (20% power, 5 s ultrasonic, 10 s interval, and 15 times repeat) was performed for Complex I enzyme activity. The UV spectrophotometer was preheated for more than 30 min, the wavelength was adjusted to 340 nm, and distilled water was adjusted to zero. Complex I activity (U/mg port) = [δA × V^total^ ÷ (ϵ × D) × 10^9^] ÷ (V^samples^ × Cpr) ÷ T = 1608 × δA ÷ Cpr Abbreviation: V^total^: Total volume of the reaction system; ϵ:NADH molar extinction coefficient = 6.22 × 103 L/mol/cm; D: Cuvette light diameter = 1cm; V^samples^: added sample volume, 0.05 ml; T: reaction time = 2 min; Cpr: sample protein concentration (mg/ml); 10^9^: unit conversion coefficient, 1 mol = 10^9^ nmol.

### ROS detectioin assay

Staining working solution (Beyotime, Shanghai, Cat. No. S0033S) was prepared as instruction. Briefly, working solutions were prepared by 1:100 dilution of 5 mM DHE solution. After the cells were routinely plated, the residual culture medium was washed with PBS thrice. After staining with working solution for 15 min, photographs were taken under a fluorescence microscope. Flow cytometry resuspend the washed cells with staining working solution and incubate at 37°C for 30 min in the dark. Use a flow cytometer for on-board detection, use 480–535 nm wavelength excitation, measure the emission above 590–610 nm, ROS-positive cells have strong red fluorescence, corresponding to the flow cytometer detection channel (BD Co. Ltd. No. Accuri C6)

### Sa-beta-Gal staining

SA-β-Gal staining was performed to detect cell senescence (14). Four groups of cell line, BEL-7404^shCtrl^, BEL-7404^shNDUFC1^, SK-HEP-1^shCtrl^, SK-HEP-1^shNDUFC1^, were cultured by 10% FBS after 72 h. After removing the culture medium, the cells were washed with PBS for three times. Add 1 ml to prepare 1 μMol/L SA- β - Gal fixative. Fix at room temperature for 15 min. After removing the stationary solution, the cells were washed with PBS for three times. Take photographs after PBS washing for three times. The BEL-7404 or SK-HEP-1 cells control were seed on plate.

### Xenograft assays

Four to 6-week-old BALB/c nude mice were used for xenograft assays. All animals were manipulated according to protocals approved by Zhejiang Medical experimental Animal Care Commission and approved by the Ethics Committee of the Zhejiang Cancer Hospital. Briefly, four groups of cell lines, BEL-7404^shCtrl^, BEL-7404^shNDUFC1^, SK-HEP-1^shCtrl^, SK-HEP-1^shNDUFC1^, were incubated separately. The serum-free medium was discarded and the cells washed once by PBS. The cells counted as 4*10^7^/ml. Twenty nude mice were randomly divided into four groups and inoculated with 1-ml cell suspension. Inoculation was performed aseptically. After inoculation, the nude mice were raised in cages, fed with water, and feed regularly, and padded materials were changed regularly. Tumor formation occurred about 1–2 weeks later. Then, the tumor size was measured every 4 days. After 30 days, the nude mice were sacrificed, photographed, and the tumors were weighed and recorded data were listed in **Supplementary Table 1**.

### Patients and tissue samples

We collected a total of 104 paraffin-embedded HCC tissue samples and paired 68 adjacent non-cancerous tissue samples from patients who were admitted to the Zhejiang Cancer Hospital between December 2012 and July 2019. The T stage was according to the 8th edition of the American Joint Committee on Cancer (AJCC) tumor-node-metastasis (TNM) staging system. Overall survival (OS) time was defined from the date of operation to the end of follow-up, or to the date of death for any reason. All experimental researches handling human cells were conducted in accordance with the Declaration of Helsinki and were approved by the Ethics Committee of the Zhejiang Cancer Hospital (Hangzhou, China). Clinical comparisons were listed in **Supplementary Table 2**.

### Immunohistochemical staining and evaluation

The formalin-fixed tumor tissue sections were deparaffinized, rehydrated, and then heated in a citric acid buffer (0.01 M) at 105°C for 10 min to recover the antigen. The sliced tissue was subsequently blocked with 3% hydrogen peroxide solution and bovine serum albumin (BSA). Then, the slides were incubated with rabbit anti-NDUFC1 (see **Supplementary Table 3** for the antibodies and dilutions used in this study) polyclonal antibody at 4°C overnight, and then incubated with horseradish peroxidase (HRP)–conjugated secondary antibody at room temperature for 20 min. The immune complexes were detected by HRP standard substrate detection. Finally, the slides were counterstained with hematoxylin, dehydrated in graded alcohol and xylene, cleared and mounted. All samples were reviewed and scored by two independent pathologists based on the intensity of staining and the percentage of positively stained cells. The intensity of staining was graded by a four-tiered scoring system (0, negative; 1, weak; 2, moderate; and 3, strong). Likewise, the cell staining positivity was assessed on a four-tiered scoring system (0, no cell stained; 1, 1–25% of cells stained; 2, 26–50% of cells stained; 3, more than 50% of cells stained). Multiply the intensity score and the percentage of positively stained cells to get the overall score. Scores <= 7 were indicative of low NDUFC1 expression, whereas scores > 7 were indicative of high NDUFC1 expression.

### Bioinformatic analysis

The RNA-SEQ data of HCC from The Cancer Genome Atlas (TCGA, USA) database were used for co-expression analysis to find the genes that might be regulated by NDUFC1. The senescence-related gene set was used to look for NDUFC1 to regulate those senescence-related genes. Only Spearman correlation ratio |r| > 0.05 and adjust *p* < 0.05 were statistically correlated (**Supplementary Table 4**).

The MSigDB dataset is a database of multiple cancer-related genes. We downloaded MSigDB (http://www.gsea-msigdb.org/gsea/index.jsp) to analysis NDUFC1 expression with cancer-related gene sets. Each cancer-related pathway score was evaluated using the GSVA algorithm. The relationship between NDUFC1 and cancer-related pathways was further analyzed. R2 > 0.25 was considered to have an interaction relationship.

### Statistical analysis

All experiments were performed in triplicate independent experiments. Data were expressed as mean ± standard deviation (*SD*) of three and more independent experiments. Statistical analyses were performed by SPSS25.0 (IBM, Armonk, NY, USA). The chi-square test and Fisher’s exact test was used to evaluate the statistical significance of the relationship between NDUFC1 protein expression and clinicopathological parameters. Student *t*-test was used to evaluated the difference of wound healing, MTT, Complex I activity data. Survival curves were assessed by Kaplan–Meier method and Cox proportional hazards regression model, and the differences were analyzed by a log-rank test. Paired Student’s *t*-test (two tails) was used for statistical analyses between two groups. Statistical significance was indicated by *p* < 0.05.

## Result

### Lentiviral shRNA inhibits the mRNA and protein expression of NDUFC1 in BEL-7404 and SK-HEP-1

**Figure 1:**
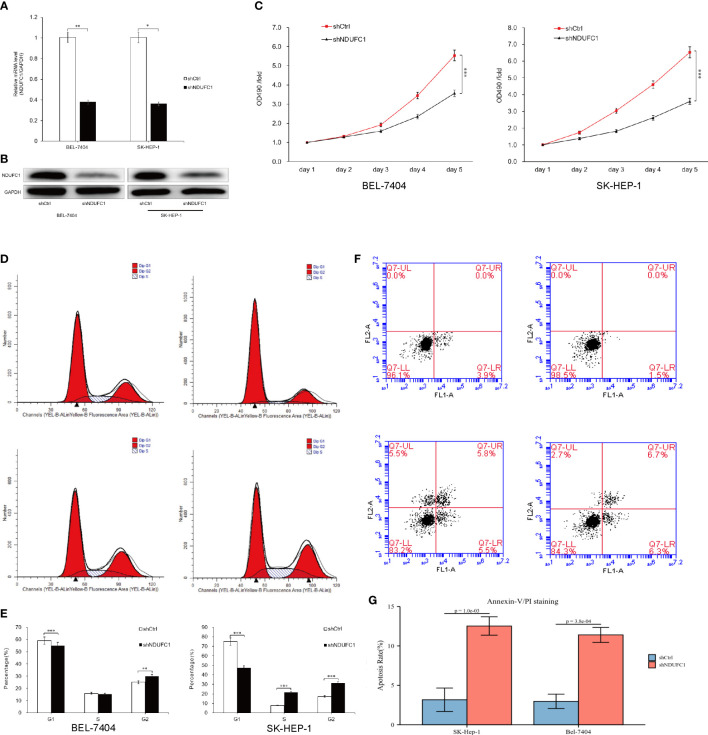
Silencing of NDUFC1 inhibited HCC cell growth. **(A, B)** qRT-PCR and Western blot results showed that NDUFC1 was stably downregulated in BEL-7404 and SK-HEP-1 cells infected with Lv-shNDUFC1. GAPDH was used as control gene. **(C)** MTT assay was used to confirm that silencing of NDUFC1 inhibits cell proliferation of BEL-7404 cells and SK-HEP-1 cells. **(D, E)** Flow cytometric analysis showed that silencing of NDUFC1 retarded the cell cycle progression of BEL-7404 cells and SK-HEP-1 cells. **(F, G)** Flow cytometric analysis was used to confirm that silencing of NDUFC1 increased apoptosis of BEL-7404 and SK-HEP-1 cells. *P<0.05; **P<0.01; ***P<0.001.

The expression of NDUFC1 messenger RNA (mRNA) in human liver cancer cell lines BEL-7404 and SK-HEP-1 was assessed by qRT-PCR, and the results indicated that NDUFC1 mRNA was highly expressed in both cell lines. In order to investigate the role of NDUFC1 in HCC, we knock down the NDUFC1 expression in BEL-7404 and SK-HEP-1 cells by Lv-shNDUFC1. The mRNA and protein expression levels of NDUFC1 in BEL-7404 and SK-HEP-1 cells were detected by qRT-PCR and Western blot to verify the knockdown efficiency. The results of qRT-PCR showed that compared with the Lv-shCtrl group, in BEL-7404 cells and SK-HEP-1 cells, the knockdown efficiency of NDUFC1 in Lv-shNDUFC1 group was 62.3% (*P* < 0.01) and 63.8% (*P* < 0.05), respectively. **(** **Figure 1A** **)** Western blot results showed that, compared with Lv-shCtrl group, the protein levels of NDUFC1 in Lv-shNDUFC1 group were also significantly decreased (**Figure 1B**).

### Silencing of NDUFC1 inhibits the proliferation of BEL-7404 and SK-HEP-1

To investigate the effect of NDUFC1 knockdown on cell proliferation, we performed a 5-day MTT assay. The result showed that Lv-shCtrl group Lv-shNDUFC1–infected BEL-7404 cells and SK-HEP-1cells showed a slower growth rate **(** **Figure 1C** **)**. The result of MTT assay revealed that NDUFC1 played an important role in the proliferation of BEL-7404 and SK-HEP-1cells. On day 5, in the BEL-7404 cells, OD490 of Lv-shNDUFC1 group was only 3.51 ± 0.12, whereas that of Lv-shCtrl group was 5.31 ± 0.10; in the SK-HEP-1 cells, OD490 of Lv-shNDUFC1 group was only 3.51 ± 0.12, whereas that of Lv-shCtrl group was 5.31 ± 0.10.

### Silencing of NDUFC1 impaired cell cycle progression of BEL-7404 and SK-HEP-1

To elucidate the impact of silencing of NDFC1 on the cell cycle progression of HCC cells, flow cytometry with PI staining was used to analyze the phases of cell cycle of BEL-7404 and SK-HEP-1 cells after NDUFC1 knockdown. As shown in **Figures 1D, E**, in BEL-7404 cells, compared with the Lv-shCtrl group, in the Lv-shNDUFC1 group, the proportion of cells in the G1 phase increased (59.04 ± 0.46% vs. 54.84 ± 0.29%, *P* < 0.001), and the proportion of cells in the G2 phase decreased (25.06 ± 0.97% vs. 29.93 ± 0.59%, *P* < 0.01). In SK-HEP-1 cells, the proportion of cells in the G1 phase of the Lv-shNDUFC1 infection group increased (75.02 ± 0.41% vs. 47.27 ± 0.20%, *P* < 0.001), and the proportion of cells in the *S* phase (7.82 ± 0.66% vs. 21.53 ± 0.38%, *P* < 0.001) and G2 phase decreased (17.16 ± 0.39% vs. 31.20 ± 0.42%, *P* < 0.001). These results indicated that the cell cycle progression was retarded in the NDUFC1 silencing HCC cells.

### Silencing of NDUFC1 induced the apoptosis of BEL-7404 and SK-HEP-1

Annexin V double staining and flow cytometric analysis were used on BEL-7404 and SK-HEP-1 cells following lentivirus infection to further to confirm the effect of NDUFC1 silencing on cell apoptosis. The results of flow cytometric analysis demonstrated that, in both BEL-7404 cells and SK-HEP-1 cells, compared with Lv-shCtrl group, apoptosis percentage increased in the Lv-shNDUFC1 infection group (*P* < 0.001, **Figures 1** **F, G**).

### Silencing of NDUFC1-reduced migration and invasion in BEL-7404 and SK-HEP-1

**Figure 2:**
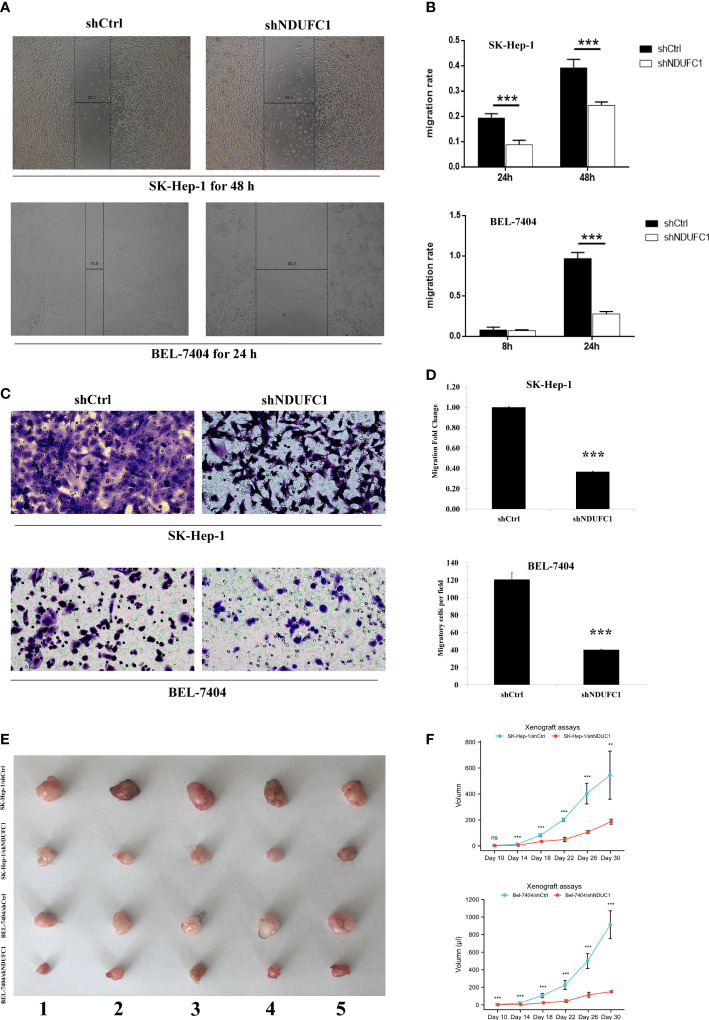
Silencing of NDUFC1 reduced migration and invasion in BEL-7404 and SK-HEP-1 cells. **(A, B)** Wound-healing assay of HCC cells transinfected with Lv-shNDUFC1 or Lv-shCtrl. **(C, D)** Transwell assay of HCC cells transinfected with Lv-shNDUFC1 or Lv-shCtrl. **P < 0.01; ***P < 0.001. ns, none significance **(E, F)**: Xenograft assays tumor formation in nude mice. Five nude mice were enrolled in the study. The tumorigenesis of shNDUFC1 group was significantly slower in SK-Hep-1 and Bel-7404.

The results of the wound-healing assay showed that, in BEL-7404 cells, after 48 h of culture, the migration rate of cells in the Lv-shNDUFC1 infection group decreased by 38% (*P* < 0.001) compared with Lv-shCtrl group **(** **Figures 2** **A, B** **)**. In SK-HEP-1 cells, after 24 h of culture, the migration rate of cells in the Lv-shNDUFC1 infection group decreased by 71% (*P* < 0.001). Transwell assay showed that compared with Lv-shCtrl group, the invasion of cells in the Lv-shNDUFC1 infection group was significantly inhibited in a time-dependent manner, with the inhibition rate at 67% (*P* < 0.01, **Figures 2** **C, D**) in BEL-7404 cells and 63% (*P* < 0.001) in SK-HEP-1. These results indicated that silencing of NDUFC1 inhibited the migration and invasion capability of the HCC cells.

### NDUFC1 could lead to tumorigenesis *in vivo*

We conducted tumorigenesis experiments in nude mice, and BEL-7404-knockout NDUFC1 group showed slow tumorigenesis 10 days after injection. The SK-HEP-1 group also began to show differences on day 14. Overall, tumor proliferation and volume were much smaller than the control group. (**Figures 2** **E, F**) Besides, according to **Figures 1** **A, B**, the knockout efficiency BEL-7404 used in our cell silencing was higher than SK-HEP-1.

### Expression of NDUFC1 in HCC and adjacent non-cancerous tissues

**Figure 3:**
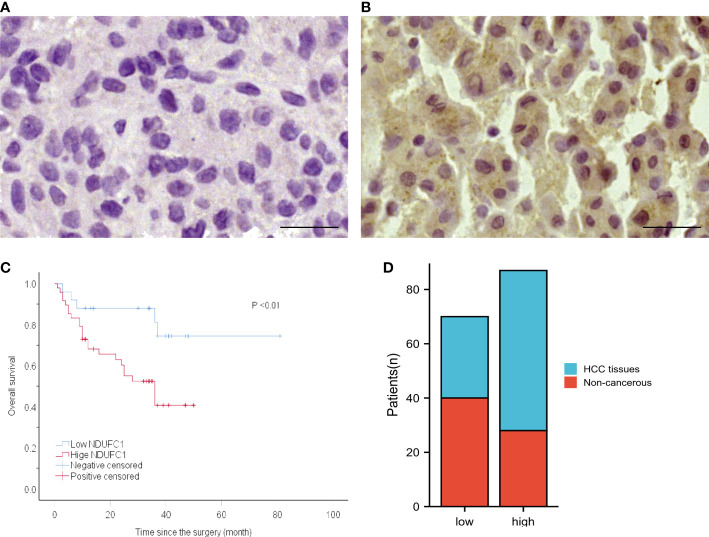
The protein expression of NDUFC1 in HCC specimens and non-cancerous tissues and the relationship between NDUFC1 expression and prognosis of patients with HCC. **(A)** NDUFC1 is negatively stained in non-cancerous tissue (left) and is positively stained in HCC specimen (right). **(B)** Kaplan–Meier survival curve shows that high NDUFC1 expression indicates a low over-all survival rate. Bar = 50 nm, **(C)** High level of NDUFC1 expression indicate poor prognosis. **(D)** NDUFC1 expression was higher in HCC.

NDUFC1 is a protein that located in the cytoplasm, so its positive performance after immunohistochemical staining is the uniform yellow staining of the cell cytoplasm or the scattered distribution of yellow stained particles in the cell cytoplasm. NDUFC1 highly expressed in 59 samples of HCC tissues, with a positive rate of 66.29% (59/89), and in 28 samples of adjacent non-cancerous liver tissues, with a positive rate of 41.18% (28/68). The high expression rate of NDUFC1 in HCC tissues was significantly higher than that in adjacent non-cancerous liver tissues (*P* < 0.01) **Figures 3** **A, B-D**.

### Survival analysis

Kaplan–Meier survival analysis revealed a significantly shorter OS in patients with high NDUFC1 expression than those with low NDUFC1 expression. **(** **Table 1** and **Figure 3C** **)** Patients with high NDUFC1 expression had a mean survival time of 22.52 ± 14.66 months, which was significantly shorter than that of patients with low NDUFC1 expression (32.60 ± 17.38, *P* < 0.05). The 1-, 3-, 5-year OS rates of patients with low NDUFC1 expression were 84.0, 48.0, and 4.0%, whereas that of patients with high NDUFC1 expression were 60.4, 14.6, and 0.0%, respectively. Compared with the low NDUFC1 expression group, patients in high NDUFC1 expression group had a shorter OS rate after surgery (HR 3.33, 95% CI 1.25 to 8.86, *P* < 0.05). The univariable and multivariable analyses results of postoperative OS in patients with HCC are shown in **Table 1**. Significant variables (*P* < 0.05) in the univariable analysis were entered into multivariable analysis, and the result identified that high NDUFC1 expression was an independent risk factor for reduced OS (HR 2.75, 95% CI 1.03 to 7.31, *P* < 0.05).

**Table 1:** Survival analysis of HCC patients.

Variable	All cases (73)	Median OS ± SD (months)	UV	UV	MV	MV
			*P* value	HR (95% CI)	*P* value	HR (95% CI)
Sex						
Male	17	20.88 ± 17.613	0.011	2.700(1.261-5.778)	0.295	
Female	56	27.52 ± 15.657				
Age						
>60	25	24.12 ± 14.692	0.317	1.466(0.693-3.099)		
<=60	48	26.9375 ± 17.079				
Smoking						
No	46	25.3 ± 17.433	0.094	2.082(0.882-4.911)	0.72	
Yes	27	27.11 ± 14.262				
Drinking						
No	55	24.80 ± 17.297	0.234	1.800(0.683-4.739)		
Yes	18	29.56 ± 12.263				
Tumor size						
<6	44	29.227 ± 16.127	0.074	1.968(0.936-4.138)	0.209	
>=6	29	21.03 ± 15.426				
LNs metastasis						
No	65	27.83 ± 15.969	3.77E-06	7.896(3.288-18.959)	7.00E-06	7.901(3.214-19.419)
Yes	8	10.88 ± 9.643				
Satellite stove						
Negative	54	27.98 ± 15.559	0.053	2.151(0.991-4.667)	0.048	2.244(1.008-4.996)
Positive	19	20.26 ± 17.243				
Vascular invasion						
Negative	56	29.45 ± 15.984	0.018	2.689(1.185-6.104)	0.425	
Positive	17	14.53± 11.364				
Nerve invasion						
Negative	61	27.8716.359	0.005	3.378(1.436-7.948)	0.271	
Positive	12	16.33 ± 12.168				
T stage						
T1+2	50	29.16 ± 16.07	0.026	2.361(1.109-5.028)		
T3+4	23	19.04 ± 14.698				
TNM stage						
I+II	48	30.06 ± 15.759	0.004	2.987(1.409-6.334)	0.748	
III+IV	25	18.12 ± 14.438				
HBs antigen						
Negative	27	26.04 ± 18.797	0.274	1.515(0.720-3.185)		
Positive	46	25.93 ± 14.782				
Margins						
Negative	67	26.90 ± 16.449	0.298	1.911(0.564-6.457)		
Positive	6	15.67 ± 9.668				
NDUFC1						
Low	25	32.60 ± 17.376	0.016	3.330(1.252-8.858)	0.043	2.747(1.032-7.307)
High	48	22.52 ± 14.662				

IHC, immunocytochemistry; LNs, lymph nodes; T, tumor; TNM, tumor-node-metastasis; HBs, hepatitis B surface; N, number; OS, overall survival; SD, standard deviation; UV, Univariable; HR, hazard ratio; CI, conﬁdence interval; MV, multivariable.

### Silencing NDUFC1 decrease mitochondrial respiratory chain Complex I activity

**Figure 4:**
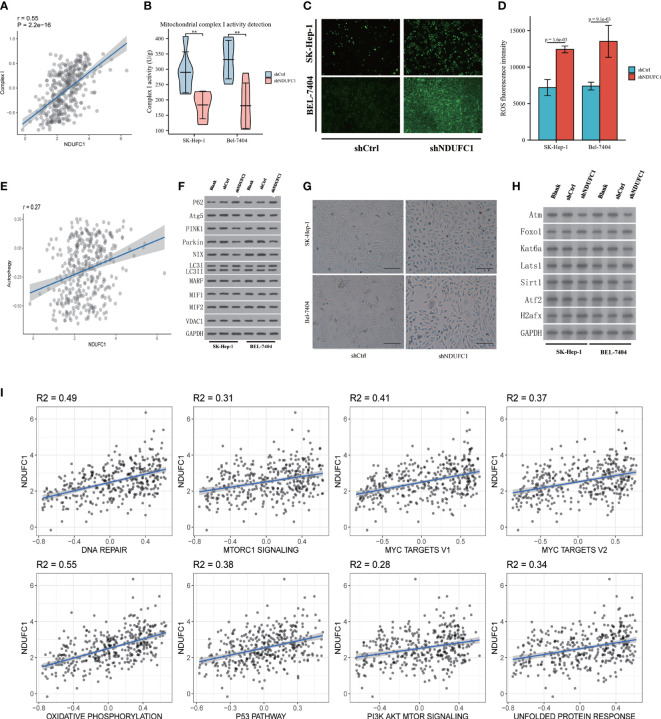
Tumor biological function of NDUFC1. **(A)** Bioinformatic analysis found positive correleation between Complex I and NDUFC1. **(B)** shNDUFC1 cell lines could inhibit the function of Complex I **(C)** ROS burden is up-regulated after NDUFC1 was knock down cell lines. **(D)** ROS flow cytometry assay showed that shNDUFC1 increased ROS burden. **(E)** Bioinformatic analysis found NDUFC1 is correlated with Autophagy. **(F)** shNDUFC1 could reduce autophagy in HCC cell lines. **(G)** SA-β-Gal analysis showed that shNDUFC1 induced senescence characteristics in SK-Hep-1 and Bel-7404. (bar = 100 μm). **(H)** Senescence related protein was correlated with NDUFC1 expression. **(I)** Cancer-associated cellular pathways analysis by NDUFC1 expression. MSigDB (http://www.gsea-msigdb.org/gsea/index.jsp) were used to analysis the cancer associated cellular pathways. The positive correlation analysis was found between NDUFC1 and multiple cancer-related pathways (R2 > 0.25 was considered as an interaction relationship). There was no negative correlation between NDUFC1 and multiple cancer-related pathways. **P < 0.01.

We measured the TCGA database and Complex I activity datasets. We found that it is positive correlated to NDUFC1 expression (*P* = 2.210^-16^, *r* = 0.55 **Figure 4A**). In order to validate the results of bioinformatics results, we measured Complex I activity in four cell lines. The results support the results of the bioinformatics analysis. The activity of Complex I decreased significantly in the SK-HEP-1^Lv-shNDUFC1^ and BEL-7404 ^Lv-shNDUFC1^ group (*P* < 0.01, **Figure 4B**).

### Silencing NDUFC1 increase reactive oxygen species

We measured the detection of ROS, whether by fluorescence microscope imaging or flow cytometry; we found that the ROS increased in the shNDUFC1 group. Both SK-HEP-1 and BEL-7404 in the knockdown group showed enhanced fluorescence. Flow cytometry also confirmed the results (*P* < 0.01). The ROS increased in the shNDUFC1 cell line (**Figures 4** **C, D**).

### NDUFC1 may inhibit autophagy

We measured the relationship between autophagy related datasets and NDUFC1 expression; we found it is positive related autophagy(*r* = 0.27 *p* < 0.001, **Figure 4E**). After collecting the protein of cell lines cultured 72 h of 10% serum, the Western blotting detection were performed. The results demonstrated that P62 increased, LC3 II/I decreased, and Atg5 decreased in the shNDUFC1 groups. It may indicate that autophagy was inhibited. In addition, the key proteins of mitophagy, PINK1, Parking, MARF, and MIF1/2 were also decreased to varying degrees. The protein VDAC1 regulated by mitochondrial dysfunction did not change significantly. Senescence-related proteins correlated with the expression NDUFC1 were also measured by Western blotting. The results have shown that Atm, Kat6a, Sirt1, and Atf2 were down-regulated, but Foxo1, Lats1, and H2afx were up-regulated in shNDUFC1 group (**Figure 4F** **).**

### NDUFC1 negative correlated to senescence

The RNA-Seq data of HCC from the TCGA database were used for co-expression analysis to find the genes that NDUFC1 might regulate. The senescence-related gene set was used to look for NDUFC1 to regulate those aging-related genes. Analysis revealed that seven aging-related genes were highly correlated with NDUFC1 expression (**Supplementart Table 4**, **Figures 4** **G, H**).

### NDUFC1 positive related to multiple cancer-associated cellular pathways

MSigDB (http://www.gsea-msigdb.org/gsea/index.jsp) was used to analyze the cancer-associated cellular pathways. A positive correlation analysis was found between NDUFC1 and multiple cancer-related pathways (R2 > 0.25 considered an interaction relationship). There was no negative correlation between NDUFC1 and multiple cancer-related pathways. Among the pathways DNA repair and Oxidative phosphorylation pathways were related to mitochondria dysfunction. We found senescence-related pathways were also involved (**Figure 4I**).

## Discussion

HCC is a second most deadly cancer in the world; patients were diagnosed at advanced stage (15). With the advancing of medical progress, treatment efficacy has greatly improved the overall survival rate of HCC patients. Current treatments for HCC including surgery, radiation, liver transplantation, and chemotherapy (16–18). But most of the death are due to lack of effective antitumor therapy (19). There is an urgent need to discover newer therapeutic targets to provide new therapeutic strategies for liver cancer treatment. The liver is responsible for the metabolism of the body and nutrients, which requires a lot of energy. This process requires mitochondria to provide energy (20, 21). Besides HCC have several known risk factor such as hepatitis infections, alcohol, and diabetes mellitus. All these risk factors are involved in metabolic disease (22). Therefore, whether the tumor metabolism of liver cancer may be different from that of other tissue types is still need clarified. Mitochondrial metabolism is closely related to the progression of HCC through a variety of mechanisms and is also a potential tumor suppressor target in clinical practice. Complex I is the largest polymerase complex in the mitochondrial respiratory chain and is responsible for the transmission of electrons and the generation of proton gradients across the mitochondrial inner membrane, thereby driving the production of ATP. In mammals, Complex I is composed of 45 subunits and must be properly assembled to form a functionally mature complex (23, 24). Seven subunits are encoded by mitochondrial DNA (mtDNA). The remaining 38 subunits are encoded by nuclear DNA (nDNA) and imported into the mitochondria from the cytoplasm (25). Among the nDNA-encoded subunits, seven subunits represent the “core subunits” in the peripheral arms of Complex I and participate in catalyzing the oxidation and electron transfer of NADH (26). The remaining 31 nDNA-encoded subunits are called “redundant” subunits and most of them do not participate in the enzymatic activity of Complex I, and their actual function is still unknown (24–26).

NDUFC1 encodes one of these “redundant” subunits of complex I. Recent studies have sporadically reported on the function of these genes (27, 28). However, the importance of the mechanism between these subunits and disease is still largely unknown. In our study, we found that NDUFC1 is highly expressed in HCC. Compared with patients with low NDUFC1 expression, patients with high NDUFC1 expression had a poor prognosis, and high NDUFC1 expression was the independent risk factor for survival. Furthermore, we found that silencing of NDUFC1 could reduce the Complex I activity and induce ROS reactive. It could further inhibit the proliferation, migration, and invasion of HCC cells and induced apoptosis and senescence.

It is known that mitochondrial Complex I participate in the regulation of Oxidative phosphorylation (OXPHOS), which is the essential metabolic on cells (29). When OXPHOS changes, it will affect the change of ROS content, which will lead to a variety of tumor biological phenotypes (23, 30, 31). NDUFC1 is the subunit that constitutes Complex I, and we suppose that it could likely involved in Complex I function in some unknown way. The results showed that Complex I activity is inhibited and increased ROS burden in BEL-7404 and SK-HEP-1. The Complex I consists of 45 subunits, some of which are involved in electron transport, some form the transmembrane structure, and some maintain function and stability (32, 33). Although we cannot fully confirm the role of NDUFC1 in Complex I, due to NDUFC1 is not located in the functional domains, it is likely to be involved in the stabilization of Complex I structure. When NDUFC1 is silenced, mitochondrial Complex I dysfunction leads to electron overflow and ROS generation. Meanwhile, NDUFC1 played a positive effect in the development of HCC in our study. A recent study found that SMIP004-7 could inhibit the growth of triple-negative breast cancer by engaging NDUFS2, which is also located in Complex I (34). Therefore, we inferred that the high expression of NDUFC1 may be involved in the tumorigenesis and lead to the bad prognosis of HCC patients.

Furthermore, in our study, flow cytometric analysis showed that silencing of NDUFC1 delayed cell cycle progression of HCC cell line, resulting in the arrest of BEL-7404 cells at G2 phase SK-HEP-1 cells at S/G2 phase. Mitochondria play a key role as an ATP generator in cell growth, and mitochondrial dysfunction may delay overall cell cycle progression *via* enhancing ROS production (35, 36). The silencing of NDUFC1 may, at least in part, allow HCC to exit the cell cycle and enter senescence status. NDUFC1 may relate to p53 pathways, DNA repair, and PI3K/Akt/mTOR pathways, which not only cancer-associated pathways but also cell death and senescence-associated pathways (37–39). However, the mechanism and specific signaling pathways of NDUFC1 involved in the biological behavior of HCC cells remain unclear. We found, for the first time, to the best of our knowledge, that NDUFC1 silencing can inactive function of Complex I and induce ROS generation. Various tumor cells are sensitive for the changes of ROS (40). Abnormal elevation of ROS in tumor cells can generate massive oxidative stress-DNA damage, mediating multiple specific programmed cell death (41, 42).

Mitochondria are potential drivers of programmed cell death, and it contributes to various ways of tumorigenesis and progression (43). Cancer cells can acquire the ability to proliferate indefinitely and avoid senescence through a variety of ways (44). Even in the absence of cytotoxicity, aging itself can mediate immune system-induced clearance and prolonged patient survival. While senescence is always combined with dysfunctional mitochondria, the changes are not yet fully understood. Tumor cellular could escape senescence during the progression of tumorigenesis. However, in the advanced stage of cancer, inducing senescence could function as profound anti-tumor effects (45). Immune cells, such as NK cells, could invade into the tumor microenvironment by SASP-mediate immune killing function and lead to long-lasting systemic anti-tumor memory response (46). Therefore, inducing senescence phenotype could facilitate anti-tumor function but without cytotoxicity. In our study, we found that silence NDUFC1 may inhibit the function of Complex I and induce ROS in HCC and could mediate the serious antitumor progression and senescence through various cancer related pathways. NDUFC1 may serve as a target for further intervention and anti-tumor effect.

Various tumorigenesis and senescence pathways were correlated with the expression level of NDUFC1. It has been reported that mitochondrial Complex I inhibitors can inhibit the proliferation of the HCC cell lines (47). Diabetes medication Canagliflozin could reduce cancer cell proliferation by inhibiting respiration supported by mitochondrial Complex I (48). Another study reported that mitochondrial Complex I inhibitor rotenone can induce cell apoptosis by enhancing the production of mitochondrial reactive oxygen species (ROS) (49). Senescence is a phenotype by which cells escape death but is associated with an inability to properly scavenge DNA damage caused by ROS (50). The immortal property of tumor cells first requires that tumors be able to escape cellular senescence by inactive DNA repair pathways (51). Senescence that prevents tumor development (52, 53). Our results consist with NDUFC1 could be a trigger for reducing ROS burden.

Our study also has several limitations. First, we have discovered that NDUFC1 is involved in regulating cancer-associated pathways and characteristics but lacks intervention in pathways. Secondly, the sample size of our study is relatively small. Thus, further large-sample and multi-center studies are needed to determine the role of NDUFC1 in the development of HCC. Last, we did not uncover the exact effect of NDUFC1 on Complex I. Therefore, future research is necessary. Notwithstanding, we still believe NDUFC1 could be a target for tumor progression by inhibiting senescence and affecting Complex I–induced mitochondrial metabolism in HCC. We suppose NDUFC1 could be a target for therapy for HCC.

## Data availability statement

The original contributions presented in the study are included in the article/**Supplementary Material**. Further inquiries can be directed to the corresponding author.

## Ethics statement

This study was reviewed and approved by IRB-2021-234. The patients/participants provided their written informed consent to participate in this study. The animal study was reviewed and approved by IRB-2021-234.

## Author contributions

Conceptualization, YZ and XC. Methodology, JL, FH, and JW. Software and validation, FH and HC. Resources and founding, FH, YZ, and XC. Data generation and quality control, DC, FH. Clinical data and follow-up, AG, WC, and YX. Writing, HC (original draft). FH (editing) manuscript was approved by all the co-authors. Supervision, YZ and HF. Project administration, FH and YZ. All authors contributed to the article and approved the submitted version.

## Funding

This work was supported by the Medical and public health projects in Zhejiang Province (2022499342, FH); the Medical and public health projects in Zhejiang Province (2022505268, YZ); Diagnosis And Therapy Center of Upper Gastrointestinal Tumor (JBZX-202006, XC).

## Acknowledgments

We are also thankful to Dr. Hao Lee (The First Affiliated Hospital of China Medical University) and Dr. Zunqiang Xiao (Zhejiang Chinese Medical University) for assistance in data processing and graph drawing. Thanks to Mrs. Han for her great support to the family over the years.

## Conflict of interest

The authors declare that the research was conducted in the absence of any commercial or financial relationships that could be construed as a potential conflict of interest.

## Publisher’s note

All claims expressed in this article are solely those of the authors and do not necessarily represent those of their affiliated organizations, or those of the publisher, the editors and the reviewers. Any product that may be evaluated in this article, or claim that may be made by its manufacturer, is not guaranteed or endorsed by the publisher.
